# Cognitive and Psychosocial Outcomes of Self-Guided Executive Function Training and Low-Intensity Aerobic Exercise in Healthy Older Adults

**DOI:** 10.3389/fnagi.2020.576744

**Published:** 2020-11-19

**Authors:** Lixia Yang, Sara N. Gallant, Leanne Karyn Wilkins, Ben Dyson

**Affiliations:** ^1^Department of Psychology, Ryerson University, Toronto, ON, Canada; ^2^Leonard Davis School of Gerontology, University of Southern California, Los Angeles, CA, United States; ^3^Department of Psychology, Memorial University of Newfoundland, St. John’s, NL, Canada; ^4^Department of Psychology, University of Alberta, Edmonton, AB, Canada

**Keywords:** executive function training, aerobic exercise, executive functions, psychosocial functions, Lumosity, aging

## Abstract

**Objectives:**

Prior work has demonstrated that executive function training or physical exercise can improve older adults’ cognition. The current study takes an exploratory approach to compare the feasibility and efficacy of online executive function training and low-intensity aerobic exercise for improving cognitive and psychosocial functioning in healthy older adults.

**Method:**

Following a standard pretest-training-posttest protocol, 40 older adults (aged 65 and above) were randomly assigned to an executive function or a physical training group. A battery of cognitive and psychosocial outcome measures were administered before and after training. During the 10 weeks of self-guided training at home (25–30 min/day, 4 days/week), the executive function training group practiced a set of adaptive online executive function tasks designed by Lumos Labs, whereas the physical training group completed an adaptive Digital Video Disc (DVD)-based low-intensity aerobic exercise program.

**Results:**

Training transfer effects were limited. Relative to low-intensity aerobic exercise, executive function training yielded cognitive improvement on the 64-card Wisconsin Card Sorting Task (WCST-64), a general executive function measure. Depression and stress levels dropped following both training programs, but this could be driven by decreased stress or excitement in performing the tasks over time.

**Discussion:**

The results revealed limited cognitive benefits of the online executive function training program, specifically to a near transfer test of general executive control. Importantly, the current study supports the feasibility of home-based self-guided executive function and low-intensity physical training with healthy older adults.

## Introduction

With a rapidly aging population, there is a growing need to identify methods to attenuate age-related cognitive decline or enhance cognition in later life (Green et al., 2019). Substantial age-related declines have been observed in executive functions (i.e., high-order attention regulation skills involved in planning, flexible thinking, and self-control; Grady, 2012), which may be associated with age-related cognitive declines in processes such as working memory (i.e., the ability to temporarily store and manipulate information; Lustig et al., 2007). The literature on cognitive training is vast but inconclusive with mixed or limited results on whether it can improve cognition during aging (Simons et al., 2016). Nevertheless, prior work has revealed that older adults’ cognitive performance could be maintained or even improved by cognitive training (Kelly et al., 2014) or physical exercise (Hillman et al., 2008).

### Cognitive Training

Prior research has shown that older adults’ cognition is somewhat malleable and may benefit from either ability-specific cognitive training in fluid abilities (i.e., the ability to think and solve problems independent of learning and education) such as reasoning and processing speed (Ball et al., 2002; Yang, 2011), or by engaging in a cognitively stimulating activity/lifestyle (e.g., educational attainment, active learning of a new skill; Stine-Morrow et al., 2008; Park et al., 2014). Cognitive training has typically shown hierarchical transfer effects (Zelinski, 2009; Wilkinson and Yang, 2015; Simons et al., 2016), with greater *near* transfer to tasks that tap into the same abilities as the training tasks than *far* transfer to tasks that assess other cognitive abilities or functional domains. Although very limited, cognitive far transfer effects have been revealed in older adults from various forms of executive function training (Jaeggi et al., 2010; Webb et al., 2018). Similarly, other work has shown that cognitively stimulating/engaging lifestyles can benefit fluid abilities (Stine-Morrow et al., 2008; Park et al., 2014). A systematic review of 52 cognitive training studies has further revealed that computerized cognitive training (CCT) programs show a small but positive effect for certain cognitive domains in healthy older adults (Lampit et al., 2014). In contrast, some other studies have shown that certain forms of CCT produce little generalization to everyday cognitive skills (Melby-Lervåg et al., 2016). However, little research has examined the feasibility and efficacy of self-guided online cognitive training, a highly accessible contemporary way of learning, with advantages in progress tracking, temporal/geographical flexibility, and instant reinforcement. Although online programs such as Lumosity have been challenged for their alleged benefits to cognition (Kable et al., 2017), some past research has shown that these programs may have beneficial effects for attention and memory (Hardy et al., 2011; Ballesteros et al., 2015).

### Physical Training

Along with an established positive relationship between physical activity and cognition in humans or animals (Swain et al., 2012), prior epidemiological and intervention studies have documented the cognitive benefits of physical exercise in older adults. Epidemiological studies suggest that older adults who remain physically active are at a decreased risk for developing cognitive impairments (Younan, 2018). Intervention studies suggest that physical exercise could yield a broad range of cognitive benefits, particularly in executive functioning (Hillman et al., 2008; Smith et al., 2010). Promisingly, a systematic review showed that even low-intensity physical exercise was effective at improving physical and cognitive health for older adults (Tse et al., 2015). Although one meta-analysis showed a potential advantage of cognitive over physical training for improving executive functions in older adults (Karr et al., 2014), another meta-analysis showed equivalent cognitive benefits between cognitive and physical exercise (Hindin and Zelinski, 2012). However, the efficacy of cognitive and physical exercise interventions have not been directly compared and thus it is unknown which type of intervention would be more likely to enhance cognition in healthy older adults. The current study thus took an explorative approach to compare the feasibility of online executive function training (i.e., *Lumosity*) with low-intensity aerobic exercise for eliciting near and far transfer effects on cognitive and psychosocial functions in older adults.

### Psychosocial Benefits

Little attention has been paid to the benefits of cognitive or physical training for older adults’ psychosocial functioning (e.g., the ability to perform the activities of daily living, regulate emotion, and engage in relationships; Kelly et al., 2014). Addressing this question allows us to identify accessible and effective ways to promote older adults’ wellbeing. Results from the Advanced Cognitive Training for Independent and Vital Elderly (ACTIVE) study suggested that speed training, but not memory or reasoning training, improved self-rated health and reduced risks for depression or declining quality of life (Wolinsky et al., 2009). Physical training, including low-intensity exercise, has also been shown to alleviate depression in young-old adults (Bridle et al., 2012; Tse et al., 2015), but not in old-old adults aged 80 and above (Ansai and Rebelatto, 2015). Taken together, the psychosocial benefits of cognitive and physical training are largely understudied, and the available evidence is mixed.

### The Current Study

We explored the efficacy of an online executive function training program against a low-intensity aerobic physical exercise regime for improving cognitive and psychosocial functioning in healthy older adults. Low-intensity aerobic exercise was used considering its effectiveness (Tse et al., 2015), safety and feasibility as a self-guided home-based exercise regime for older adults. Following the hierarchical transfer taxonomy (Zelinski, 2009; Wilkinson and Yang, 2015), we examined cognitive near transfer effects against outcome measures that overlapped with the training tasks in structure/ability (i.e., executive function or working memory), cognitive far transfer effects against tasks tapping untrained cognitive abilities (i.e., speed and episodic memory), and psychosocial far transfer effects against distant tasks assessing depression, stress, anxiety, and everyday activities. In light of the cognitive (Yang, 2011; Sprague et al., 2019) and physical training literature (Hillman et al., 2008), we predicted that executive function training would lead to small but positive cognitive near transfer effects (Lampit et al., 2014) while physical training might otherwise show a broader cognitive benefit. Based on prior work (Wolinsky et al., 2009; Bridle et al., 2012), it is further predicted that both training protocols might show psychosocial benefits.

To address these goals, healthy older adults were enrolled in an executive function or a low-intensity physical exercise training program, which required the completion of self-guided activities at home for 25–30 min/day, 4 days/week for 10 weeks. Self-guided training/exercise has been shown to be effective in eliciting cognitive benefits without noted adherence problems in older adults (Yang et al., 2006; Yang, 2011; Hindin and Zelinski, 2012). The executive function training group practiced a set of online executive function or working memory tasks designed by Lumos Labs,^1^ whereas the physical training group completed an aerobic exercise program following a series of DVDs.

## Materials and Methods

### Participants

Based on *a priori* power analysis using G^∗^Power 3.1.9.2 (Faul et al., 2007), a sample of 38 participants would provide 85% power to detect the critical Group × Session interaction (which signals the transfer effect) with a medium effect size of *f* = 0.25 (corresponds to η^2^ = 0.06). The final sample included 40 healthy older adults (aged 65–87; *M* = 70.83, SD = 5.25, see Table 1) and informed consent was collected before their participation. Participants were recruited from the Ryerson Senior Participant Pool (RSPP), a university-organized database of approximately 700 older adults.

**TABLE 1:** Sample characteristics.

Variables	Executive function training (*n* = 20)	Physical training (*n* = 20)	Group difference
			*p*
Age (years)	72.25 (5.98)	69.40 (4.07)	0.086
Gender (male:female)	5:15	5:15	1.000
Formal education (years)	18.32 (2.52)	17.00 (2.70)	0.124
Health rating	8.35 (0.93)	8.75 (1.16)	0.232
MAQ (minutes)	24,687.40 (19,561.08)	31,586.20 (22,360.19)	0.306
CAQ (sum score)	39.15 (7.23)	42.90 (5.21)	0.068
MMSE	28.40 (1.70)	28.45 (1.54)	0.923
Home step test (number of steps)	53.53 (9.25)	61.00 (13.68)	0.069
Home step test (heart rate increase)	47.33 (19.78)	50.32 (17.81)	0.632

**Most cells present the mean (M), with standard deviation (SD) in parenthesis, except for gender cells which present a ratio score. Between-group comparisons were made using separate independent t-tests apart from gender ratio, which was examined using Pearson’s chi square. MAQ, the Modifiable Activity Questionnaire; CAQ, the Cognitive Activity Questionnaire; MMSE, the Mini-Mental State Examination. Sample size = 40 for all the comparisons, except for education (n = 39), number of steps (n = 35), and heart rate increase (n = 37). Heart rate was assessed in beats per minute (BPM) and the heart rate increase was calculated by subtracting the baseline BPM from the BPM right after the Home Step Test*.*

They were first screened for eligibility through their database information and via phone screening to include those without: (1) severe medical conditions (e.g., uncontrolled diabetes and/or cholesterol, cardiovascular diseases) that might endanger their participation in physical fitness training; (2) previous neurological disorders including stroke, prolonged periods of unconsciousness, and head injury; (3) uncorrected vision or hearing problems; and (4) previous participation in a cognitive or physical training intervention within the past five years. Participants were also required to have access to the internet or a DVD player as well as prior experience navigating the internet. Participants that met these criteria were invited to participate and randomly assigned to the executive function or the physical training group. At the pretest session, participants were further screened for potential dementia-related cognitive impairment using the Mini-Mental Status Exam (MMSE; Folstein et al., 1975), and all of them scored above the cut-off score of 23. Those assigned to the physical training group also completed the Physical Activity Readiness Questionnaire for Everyone (PAR-Q +; Warburton et al., 2011). Based on their responses, those with health and medical conditions (*n* = 6) were asked to consult with a qualified health care practitioner or exercise professional for potential health and safety issues related to their participation before they start.

At pretest, all participants completed (1) the Modifiable Activity Questionnaire (MAQ; Kriska et al., 1997) for self-reported time in 40 physical activities over one month; (2) a lab-made Cognitive Activity Questionnaire (CAQ) for self-rated frequency of engagement in 12 cognitively stimulating activities using a 5-point Likert Scale, with ‘1’ indicating ‘once a year or less’ and ‘5’ meaning ‘every day or about every day’; and (3) the Home Step Test (CSTF, 2020) for physical fitness, which requires stepping up and down on an exercise step for 3 min while heart rate is being recorded at baseline and immediately after. The two groups were similar on these variables (see Table 1).

### Design and Procedure

This study adopted a standard pretest-training-posttest protocol using a 2 (training: executive function vs. physical) × 2 (session: pretest vs. posttest) mixed-model design, with training group as a between-subjects variable and session as a within-subjects variable.

#### Pretest and Posttest Sessions

A battery of cognitive and psychosocial outcome measures (Table 2) were administered at both the pretest and posttest sessions (approximately 3.5–5 hours each), within a week of the start and the completion date of the 10-week training schedule, respectively.

**TABLE 2:** Summary of pretest vs. posttest performance, transfer effects, and test-retest correlations on transfer tasks.

Measures		Executive function training		Physical training		Group × session interaction			*r*
				
		Pretest *M* (SD)	Posttest *M* (SD)	Pretest *M* (SD)	Posttest *M* (SD)	*P*	η_p_^2^	*BF*	
**Cognitive near transfer: *N*-back**									
	0-back hit	0.98 (0.03)	0.97 (0.10)	0.94 (0.19)	1.00 (0.02)	0.140	0.06	0.75	–0.08
	1-back hit^s^	0.83 (0.17)	0.93 (0.10)	0.86 (0.17)	0.92 (0.08)	0.547	0.01	0.36	0.41**
	2-back hit	0.71 (0.18)	0.79 (0.12)	0.81 (0.17)	0.79 (0.15)	0.052	0.10	1.47	0.55**
	0-back false alarm	0.01 (0.03)	0.02 (0.04)	0.03 (0.05)	0.01 (0.03)	0.123	0.06	0.82	0.34*
	1-back false alarm^s^	0.05 (0.09)	0.04 (0.05)	0.06 (0.07)	0.03 (0.04)	0.402	0.01	0.36	0.70**
	2-back false alarm	0.09 (0.05)	0.10 (0.05)	0.09 (0.07)	0.06 (0.04)	0.134	0.06	0.78	0.48**
**Cognitive near transfer: Stroop**									
	Accuracy interference^g^	0.89 (0.23)	0.95 (0.11)	0.98 (0.03)	0.99 (0.03)	0.339	0.02	0.45	0.40*
	RT interference (ms)	1.26 (0.12)	1.27 (0.18)	1.20 (0.10)	1.21 (0.12)	0.960	< 0.01	0.31	0.53**
**Cognitive near transfer: Navon**									
	Local accuracy interference	0.16 (0.33)	0.03 (0.27)	0.04 (0.10)	0.06 (0.14)	0.199	0.04	0.61	−0.35*
	Global accuracy interference^s^	0.25 (0.28)	0.11 (0.11)	0.15 (0.11)	0.14 (0.20)	0.058	0.09	1.36	0.29
	Local RT interference (ms)	92.87 (164.53)	29.44 (387.36)	99.99 (104.97)	246.52 (863.53)	0.337	0.02	0.45	0.01
	Global RT interference (ms)	247.82 (269.73)	220.67 (274.64)	219.63 (269.8)	574.74 (128.52)	0.452	0.02	0.39	0.39*
**Cognitive near transfer: WCST-64**									
	Total correct^gs^	38.75 (10.98)	45.35 (10.17)	46.05 (9.29)	44.10 (11.59)	0.001**	0.25	32.47	0.68**
	Perseverative responses	13.80 (8.36)	10.70 (8.05)	9.25 (7.48)	11.00 (6.76)	0.067	0.09	1.23	0.42**
	Perseverative errors^gs^	12.40 (7.28)	9.50 (6.17)	8.40(5.99)	9.70 (5.65)	0.038*	0.11	1.82	0.49**
	Non-perseverative errors^gs^	12.85 (5.94)	9.15 (4.77)	9.55 (4.89)	10.20 (7.12)	0.020*	0.13	2.94	0.47**
	Conceptual level responses^gs^	31.25 (14.96)	39.60 (14.84)	41.20 (13.00)	38.80 (15.57)	0.016**	0.18	7.17	0.63**
	Categories completed^gs^	2.05 (1.57)	2.65 (1.53)	2.85 (1.57)	2.65 (1.76)	0.044*	0.10	1.65	0.69**
	Trials to complete first category^gs^	27.65 (20.61)	18.35 (15.89)	18.70 (12.51)	26.15 (21.58)	0.012*	0.16	4.32	0.31
	Failure to maintain set	0.35 (0.67)	0.60 (1.10)	0.75 (0.97)	0.45 (0.60)	0.098	0.07	0.95	0.26
	Learning to learn	1.68 (7.87)	−4.37 (6.43)	0.85 (7.30)	−1.51 (9.87)	0.963	< 0.01	0.42	0.09
**Cognitive near transfer: change-detection**									
	1-target^s^	0.81 (0.11)	0.86 (0.12)	0.81 (0.11)	0.84 (0.10)	0.334	0.03	0.65	0.82**
	1-target 2-distractor^s, gs^	0.80 (0.11)	0.86 (0.11)	0.81 (0.12)	0.83 (0.10)	0.042*	0.11	1.71	0.78**
	3-target	0.66 (0.08)	0.68 (0.09)	0.66 (0.09)	0.67 (0.09)	0.469	0.01	0.57	0.76**
**Cognitive far transfer: DSST**									
	# Correct solutions^g^	59.15 (13.74)	59.35 (14.34)	69.45 (17.86)	70.95 (16.70)	0.478	0.01	0.38	0.94**
**Cognitive far transfer: HVLT-R**									
	Immediate recall	24.15 (5.04)	25.20 (6.65)	25.60 (4.74)	26.00 (5.70)	0.688	< 0.01	0.33	0.60**
	Recall learning slope	1.55 (0.79)	1.48 (1.02)	1.75 (0.87)	1.55 (0.84)	0.702	< 0.01	0.33	0.33*
	Delayed recall	8.05 (2.31)	8.85 (2.30)	9.15 (1.66)	9.20 (2.46)	0.313	0.01	0.47	0.46**
	Retention	0.84 (0.18)	0.89 (0.13)	0.91 (0.13)	0.90 (0.15)	0.288	0.03	0.49	0.24
	Recognition discrimination^g^	10.75 (1.41)	10.95 (1.50)	11.35 (0.81)	11.42 (0.69)	0.770	< 0.01	0.32	0.16
**Psychosocial far transfer: DASS-21**									
	Depression^g,s^	6.70 (8.34)	4.10 (6.60)	2.30 (2.92)	1.60 (3.15)	0.181	0.05	0.64	0.74**
	Anxiety	5.00 (6.10)	5.40 (6.68)	2.50 (5.10)	1.65 (4.02)	0.286	0.03	0.49	0.80**
	Stress^g,s^	11.00 (7.09)	9.40 (6.90)	5.60 (5.37)	3.50 (4.44)	0.709	< 0.01	0.33	0.80**
**Psychosocial far transfer: IADL**									
	Sum score	7.80 (0.52)	7.80 (0.92)	7.70 (0.73)	7.80 (0.41)	0.423	0.02	−	0.79**

WCST-64, 64-card Wisconsin card sorting task; DSST, Digit Symbol Substitution Task; HVLT-R, Hopkins Verbal Learning Test-Revised; DASS-21, 21-item depression anxiety stress scales; IADL, instrumental activities of daily living scale. p and η_p_^2^ values are for the Group × Session interaction that signals the transfer effects. BF, Bayes factor (not calculated for IADL due to lack of variance at baseline). ^g^Group effect was significant; ^s^Session effect was significant; ^gs^Group × Session interaction was significant; *p < 0.05, **p < 0.01.

#### Training Sessions

Similar to previous work (Berryman et al., 2014), we adopted a short-term training schedule (i.e., 25–30 min/day, 4 days/week for 10 weeks). At each training session, the executive function group completed an online cognitive training course consisting of 10 Lumosity executive function tasks, which included five for the first 10 sessions, and then added one new task every 5 sessions (see Supplementary Appendix A for task descriptions). All tasks were adaptive to participants’ individual performance level. At each session, the physical group completed an indoor aerobic exercise workout following a video clip from one of three DVD workout programs, which featured low-intensity exercise appropriate for older adults to do on their own: *Jane Fonda’s Prime Time*, *Winnipeg in Motion*, and *Jane Fonda’s Firm and Burn* representing easy, medium, and difficult intensity levels, respectively. Participants were instructed to start with the easy program and gradually progress to higher intensity levels based on their own performance and fitness level, targeting a minimum of 50% heart rate increase at each session. These DVD programs were selected considering their popularity and focus on low-impact aerobic exercise (Krucoff, 1990). The clips were reviewed, piloted, and selected by the research team to ensure age-appropriateness, safety, length, and feasibility for home-based exercise. Each session started with a brief warm-up period, followed by 25–30 min of aerobic exercise, and then by a cool-down period. Participants were given a heart-rate monitor watch to record their heart rate right before the warm-up (baseline), and after the aerobic exercise section but prior to cool-down (post-exercise) at each session.

A training log was also completed at each training session to record time, heart rate readings, and note any problems or general comments on the training tasks. The log also included weekly activity tracking in which participants recorded the time (in minutes) they spent in various cognitive (e.g., reading, writing, gaming) or physical (e.g., jogging, swimming, dancing) activities outside the training program. To check on progress and address questions, participants were called three times a week. Training completion was monitored through Lumos Labs’ data, heart rate recordings, and training logs. Based on the daily training logs, the average adherence rate (i.e., percentage of sessions completed) was 93.88% (91.63% for the executive function group and 96.13% for the physical group). More than 80% of participants completed over 90% of the training sessions.

### Outcome Measures

#### Cognitive Near Transfer Tasks

Cognitive near transfer tasks included those that were structurally similar to or taxed the same abilities (e.g., executive function or working memory) as the training tasks (Supplementary Material: Appendix A).

##### The Digit N-Back Task

The digit *N-Back task* (Wilkinson and Yang, 2016a) is an executive function task that taps into updating abilities. Participants viewed sequentially presented single digits (1–9) and indicated via key press whether each digit matched a pre-specified target (0-back), the digit presented immediately before (1-back), or two trials before (2-back). There were three blocks of trials, each including 10 practice trials followed by 45 test trials (including 9 target trials). Participants pressed the z key, labeled as “TARGET,” and the/key, labeled as “NON-TARGET,” counterbalanced across participants, as fast and accurately as possible. Two parallel versions of the task were counterbalanced across the pretest and the posttest sessions, with different digit sequences and target stimuli (i.e., 5 or 7 in the 0-back block). The dependent variables included hit rate (i.e., the proportion of targets correctly identified), false alarm (FA) rate (i.e., the proportion of non-targets misidentified as targets), and reaction time (RT).

##### The Stroop Task

The *Stroop task* (Stroop, 1935; Wilkinson and Yang, 2016b) is an executive function task that utilizes inhibition. Participants viewed single words and indicated the ink color of the word by pressing corresponding keys on the keyboard as fast and accurately as possible. They completed 280 trials (including 64 practice trials), which included an equal proportion of three trial types: congruent (e.g., the word “BLUE” printed in blue ink), incongruent (e.g., the word “BLUE” printed in green ink), and neutral (e.g., “XXXX” printed in blue ink). Two parallel versions of the task were counterbalanced across the pretest and the posttest sessions, using different sets of colors (green, purple, blue, and orange in Set 1 and orange, yellow, pink, and green in Set 2). Following previous practice (Wilkinson and Yang, 2016a), the dependent variable was the Stroop interference ratio score, calculated by dividing mean RTs or accuracy (i.e., hit rate) of incongruent trials by that of neutral trials (i.e., RT interference ratio score = RT_incongruent_/RT_neutral_; Accuracy interference ratio score = Hit_incongruent_/Hit_neutral_).

##### The Navon Task

The *Navon task* (Navon, 1977) is an executive function task that utilizes response switching and interference resolution. Participants responded to the global or the local features of a series of compound letter stimuli. Following 16 practice trials, there were two blocks of 144 test trials (72 local and 72 global), which included two trial types: congruent trials with the two local and global dimensions matched (e.g., a large letter H composed of small letter Hs) and incongruent trials with the two dimensions mismatched (e.g., a large letter S composed of small letter Hs). Global and local trial types were intermixed within blocks and thus participants needed to switch the response dimensions from trial to trial. At the start of each trial, a cue signaled which feature to respond to, with a large rectangle cueing a global response and a small rectangle cueing a local response trial. Two versions of the task, with different letter stimuli (“S” and “H” or “A” and “E”), were counterbalanced across the pretest and the posttest sessions. Participants indicated which letter (e.g., “S” or “H”) was the target letter at a global or local dimension by pressing the corresponding keys (z or/), as fast and accurately as possible. The key assignment was counterbalanced across participants but kept consistent between pretest and posttest sessions. Following prior practice (Wilkinson and Yang, 2016a), interference scores were calculated as the difference between congruent and incongruent trials in both RT and errors for each dimension (i.e., RT interference = RT_incongruent_ − RT_congruent_; Accuracy interference = Error_incongruent_ − Error_congruent_).

##### The Computerized 64-Card Wisconsin Card Sorting Task

The computerized *64-card Wisconsin Card Sorting Task* (WCST-64; Kongs et al., 2000) assesses general executive control (planning, reasoning, set switching, flexible thinking, and updating, etc.). Across 64 trials, participants matched a response card to one of four stimulus cards based on one of the three sorting rules (color, shape, or number). Responses were made by pressing the number keys (1–4), each corresponding to one of the stimulus cards. Participants were not informed of the correct sorting rule or when the rule shifted. The sorting rule was inferred via feedback (“Right” or “Wrong”) following each response. Performance was indexed by nine variables: (1) *total correct* refers to the number of correct trials; (2) *perseverative responses* are cards continuously sorted, regardless of accuracy, according to a specific rule; (3) *perseverative errors* are cards continuously sorted according to a previous rule even after the rule has changed; (4) *non-perseverative errors* are other errors; (5) *conceptual level responses* are instances of three or more consecutive correct responses; (6) *categories completed* are instances of 10 consecutive correct responses; (7) *trials to complete the first category* are trials needed to successfully complete the first category; (8) *failure to maintain set* is the number of failures to continuously respond based on a correct sorting rule; and (9) *learning to learn* refers to the change in errors between successive categories.

##### The Change-Detection Task

The *Change-Detection task* (Jost et al., 2011) is a working memory task (i.e., capacity and distraction regulation specifically) in which participants were instructed to remember the orientation of target items (red rectangles) and ignore distracters (blue or green rectangles), that were presented as an array on either the left or right side of the screen. There were 120 trials equally split across three trials types, including “1-target,” “3-target,” and “1-target plus 2-distractors.” Each trial began with an arrow cue directing participants to attend to the left or the right side of the screen. Following the testing stimulus array, a probe rectangle was presented and participants indicated whether the orientation of the probe was the same as the target item at the cued location by pressing the z or/keys, labeled as “yes” or “no,” as fast and accurately as possible. The response key assignment was counterbalanced across participants. Performance was indexed by accuracy (i.e., percentage of correct responses) and RT.

#### Cognitive Far Transfer Tasks

Cognitive far transfer tasks assessed cognitive abilities that were not practiced during training. This included the *Digit Symbol Substitution Test* (*DSST*; Wechsler, 1981), a processing speed task in which participants substituted as many digits as possible with their corresponding symbols according to a provided digit-symbol mapping key. Participants first completed 7 practice trials followed by 133 trials in 2 min. The dependent variable was the number of correct completions. The *Hopkins Verbal Learning Test-Revised* (*HVLT-R*; Benedict and Hopkins, 2020) was used to assess verbal learning and memory. Participants learned 12 nouns from three semantic categories, followed by three trials of immediate recall (Trials 1–3). After 20 min, there was a delayed recall (Trial 4) and a yes/no recognition test including 12 lures (Trial 5). There were five dependent variables: (1) *total immediate recall* across Trials 1–3; (2) *immediate recall learning slope* (average gains per trial across Trials 1–3); (3) *delayed recall* (recall at Trial 4); (4) *retention* (Trial 4 divided by Trial 2 or 3, whichever was higher); and (5) *recognition discrimination* (hits minus FAs on Trial 5). No ceiling effects were noted (Table 2). Each of these tasks had two parallel versions, counterbalanced across the pretest and the posttest sessions.

#### Psychosocial Far Transfer Tasks

The *Depression Anxiety Stress Scale* (DASS-21; Lovibond and Lovibond, 1995) was used to assess depression, anxiety, and stress during the past week, which has a test-retest reliability ranging from 0.81–0.88 across the three subscales (Osman et al., 2012). Participants rated seven statements for each subscale using a Likert scale ranging from 0 (“did not apply to me at all”) to 3 (“applied to me very much or most of the time”). Each of the depression, anxiety, and stress subscales were indexed by its own summed score, multiplied by 2. The *Instrumental Activities of Daily Living* (IADL) scale was used to assess functioning in eight daily living activities (i.e., ability to use telephone, shopping, food preparation, housekeeping, laundry, mode of transportation, responsibility for own medications, ability to handle finances), with a test-retest reliability of 0.80 (Lawton and Brody, 1969). For each activity, participants selected from a list of statements the one that most closely described their current level of functional ability (e.g., “Does personal laundry completely”). Each item was scored “1” if the ability could be performed at some minimal level of functioning or higher, otherwise, it was scored “0.” The dependent variable was the total score, with a lower score indicating a higher dependence level.

### Statistical Analysis

Data were analyzed in IBM SPSS 24. Significance level was defined at *a* = 0.050. Where necessary, Bonferroni corrections were modeled into the analyses to correct p-values for any exploratory multiple comparisons. Practice effects for each training task were assessed with a linear regression model for progressive changes in performance on executive function tasks or activity intensity of physical exercise across practice sessions. To test transfer effects, each dependent variable of the outcome measures was submitted to a 2 (Group: executive function vs. physical) × 2 (Session: pretest vs. posttest) mixed-model analysis of variance (ANOVA), with Group as a between-subjects variable and Session as a within-subjects variable. The transfer effect was indexed by the Group × Session interaction, with each group serving as a control for the other. To control for group baseline differences, variables/conditions that showed transfer effects were further analyzed on proportional training gain scores (gain from pretest to posttest divided by pretest). One-way ANOVAs were used to test group differences in training gain and one-sample *t*-tests followed to assess whether each group showed significant performance gains (i.e., above zero) at posttest compared to pretest baseline. The interaction (i.e., transfer effect) for each variable was further tested for robustness with Bayesian hypothesis testing in Jeffreys’s Amazing Statistics Program (JASP) (Van Doorn et al., 2019). Bayesian analysis confirms the likelihood of the presence or absence of an effect (alternative vs. null hypothesis) as indexed by a Bayesian factor (BF), with a BF = 3–10 meaning a moderate effect, and a BF over 10 suggesting a strong effect.

## Results

Eight participants (two in the executive function and six in the physical group) dropped out due to time restraints and were then replaced. Based on the independent-sample *t*-tests, no attrition effect was detected in most demographic (i.e., age, education, health rating, MAQ and CAQ scores) and psychosocial variables (i.e., DASS-21 and IADL scores), as well as on the DSST and MMSE, *p*s ≥ 0.173. They also did not statistically differ from the final sample in the number of steps, immediate recall, recall slope, and retention (*p*s ≥ 0.058). However, drop-out participants did show slightly different physical and memory profiles relative to the final sample as evidenced by their lower heart rate increase after the Home Step Test (*p* = 0.011) and lower recognition discrimination in HVLT (*p* = 0.023).

### Practice Effects

Executive function training task performance was indexed by the Lumosity Performance Index (LPI), a standardized score generated and recorded by the Lumos Lab’s Server after each training session. Exercise intensity at each session was indexed by the heart rate reserve (HRR), the proportional increase of peak (post-exercise) relative to resting (baseline) heart rate, as assessed in beats per minute (BPM; Smith et al., 2010). To assess practice effects, a linear regression model was conducted with session number as the predictor and LPI/HRR as the outcome variable for each training task (Table 3). All executive function training tasks showed significant practice effects, with no apparent ceiling effect (*R*^2^s ≥ 0.73, βs ≥ 4.14, *p*s ≤ 0.01), but HRR did not show significant practice effect (*p* = 0.65). The average HRR was 40.21%, suggesting a light exercise intensity (Kramer et al., 2002).

**TABLE 3:** Linear regressions on the prediction of session number to training performance.

	# of Sessions	Initial performance	Final performance	*R*^2^	*F*	*B*	*p*
**Executive function training**							
Brain shift	40	310.60	863.8	0.97	1,055.39	13.27	< 0.001
Color match	40	300.00	729.38	0.96	903.07	10.86	< 0.001
Face memory workout	40	216.20	456.87	0.98	1,627.60	5.88	< 0.001
Lost in migration	40	311.75	630.00	0.96	965.27	7.38	< 0.001
Memory matrix	40	425.45	638.15	0.93	499.60	4.15	< 0.001
Disillusion	30	291.05	784.87	0.98	1,478.31	17.64	< 0.001
Follow the frog	25	264.56	525.71	0.96	540.31	10.07	< 0.001
Route to sprout	20	242.06	702.79	0.80	73.06	17.82	< 0.001
Observation tower	15	412.10	603.47	0.68	27.51	8.86	< 0.001
Pinball recall	10	301.06	485.86	0.73	21.69	15.94	= 0.002
Physical training	40	30.51	40.57	0.01	0.20	0.02	0.65

**Executive function training task performance was indexed by Lumosity Performance Index (LPI). Physical exercise performance was indexed by heart rate reserve (HRR). Please see Supplementary Appendix A for the detailed description of each executive function training task*.*

### Transfer Effects

Prior to analyzing the RT data, outliers were trimmed by removing trials that were ± 2.5 SDs from the mean within each condition. Initial analyses on RTs in the *N-Back* and the *Change-Detection* tasks did not reveal a significant interaction (*F*s ≤ 2.46, *p*s ≥ 0.126), thus they were omitted for brevity. All transfer effects (i.e., the Group × Session interaction and its BF) and reliability (pretest-posttest correlations) for each dependent variable are reported in Table 2.

#### Cognitive Near Transfer

For the N-Back task, the ANOVAs on hit and FA rates for each condition revealed significant Session effects for the 1-back condition in both hit, *F*(1,38) = 10.93, *p* = 0.002, η_p_^2^ = 0.22, and FA rates, *F*(1,38) = 5.85, *p* = 0.020, η_p_^2^ = 0.13, with a higher hit and lower FA rate at posttest vs. pretest, indicating improvement in both indices. The critical Group × Session interactions were not significant (*p*s ≥ 0.052).

The ANOVAs on the Stroop RT and accuracy interference scores revealed larger interference in accuracy for the physical (*M* = 0.99, SD = 0.03) than the executive function group (*M* = 0.92, SD = 0.14), *F*(1,38) = 4.24, *p* = 0.046, η_p_^2^ = 0.10; all other effects were non-significant (*p*s ≥ 0.111). For the Navon task, the ANOVAs on RT and accuracy interference scores for each condition revealed a significant Session effect in the accuracy analysis for the global condition, *F*(1,38) = 4.49, *p* = 0.041, η_p_^2^ = 0.11, with reduced interference scores at posttest relative to pretest (i.e., improvement), but the critical Group × Session interaction did not reach significance (*p* = 0.058). The local condition analyses did not reveal any significant effects (*p*s ≥ 0.199).

**FIGURE 1:**
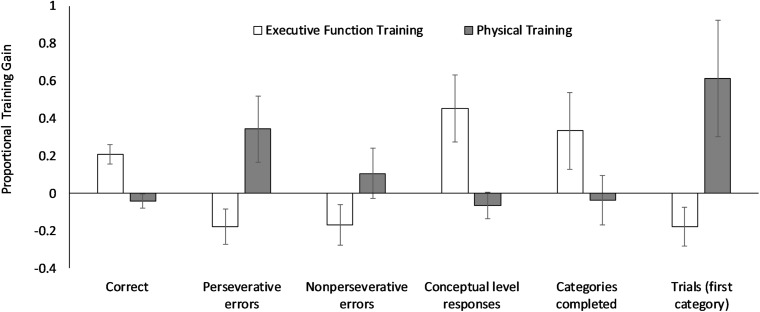
The proportional training gain scores in the 64-card Wisconsin Card Sorting Task (WCST-64). Positive values mean the proportional score increases at posttest relative to pretest, with higher values meaning larger gains. Error bars refer to the standard errors of the means. Trials (first category) = trials to complete the first category.

For the WCST-64 task, the ANOVAs revealed a significant Group × Session interaction for all dependent variables, *F*s ≥ 4.34, *p*s ≤ 0.044, η_p_^2^s ≥ 0.10, except for perseverative responses (*p* = 0.067), failure to maintain set (*p* = 0.098), and learning to learn (*p* = 0.963). One-way ANOVAs on the proportional gain scores (Figure 1) confirmed the group differences in total correct, perseverative errors, conceptual level responses, and trials to complete the first category (*F*s ≥ 5.75, *p*s ≤ 0.021, *d*s ≥ 0.76). The follow-up one-sample *t-*tests showed significant gains in total correct (*t* = 3.90, *p* = 0.001, *d* = 0.87) and conceptual level responses (*t* = 2.52, *p* = 0.021, *d* = 0.57) but not in other variables (*p*s ≥ 0.078) following the executive function training. In contrast, physical training did not produce any significant gains (*p*s ≥ 0.064). As a further validation, these variables were also analyzed by artificially matching the samples based on the baseline “total correct” range (i.e., 36–55), resulting in 12 participants in the executive function training and 17 in the physical training group, without group differences in all dependent variables (*p*s ≥ 0.46). The one-way ANOVAs on the proportional gain scores of these baseline-matched samples revealed significant group differences in the following variables: total correct, conceptual level responses, and perseverative errors (*p*s ≤ 0.05). The one-sample *t*-tests showed significant training gains in both total correct and conceptual level responses (*p*s = 0.02) for the executive function training group, whereas the physical group did not show significant gains in any variables (*p*s ≥ 0.12).

For the Change-Detection task, we excluded one executive function training participant due to low accuracy at pretest (0.07–0.23 correct across conditions). The ANOVAs on accuracy revealed significant pretest to posttest improvement for the “1-target” and the “1-target plus 2-distractors” conditions, *F*s ≥ 12.26; *p*s ≤ 0.001, η_p_^2^s ≥ 0.24, but not for the “3-target” condition (*p* = 0.191). The Group × Session interaction was significant only for the “1-target plus 2-distractors” condition, *F*(1,37) = 4.44, *p* = 0.024, η_p_^2^ = 0.11, with a significant benefit from the executive function training, *t(*18) = −3.56, *p* = 0.002, *d* = 0.77, but not the physical training, *t(*19) = −1.12, *p* = 0.278, *d* = 0.33. The one-way ANOVAs on the proportional gain score failed to reveal a significant group difference (*p* = 0.083). The one-sample *t*-tests showed significant gains in the “1-target” condition for both groups (*t*s ≥ 2.74, *p*s ≤ 0.013, *d*s ≥ 0.62), in the “1-target plus 2-distractors” condition for the executive function group, *t(*18) = 3.49, *p* = 0.003, *d* = 0.76, but not the physical group (*p* = 0.182). Neither groups benefited in the “3-target” condition (*p*s ≥ 0.165).

#### Cognitive Far Transfer

The ANOVA on the total number of correct responses on the DSST revealed a significant effect of Group, *F*(1,38) = 5.00, *p* = 0.031, η_p_^2^ = 0.12, with a higher score in the physical (*M* = 70.20, *SD* = 17.10) than the executive function group (*M* = 59.25, SD = 13.69), *d* = 0.71. All other effects were not significant (*p*s ≥ 0.354).

For the HVLT-R, the ANOVAs on the five dependent variables outlined in the Methods section did not reveal any significant effects (*p*s ≥ 0.060).

#### Psychosocial Far Transfer

For the DASS-21, the ANOVAs on the depression, anxiety, and stress scores revealed a significant main effect of Group (*F*s ≥ 4.24, *p*s ≤ 0.046, η_p_^2^s ≥ 0.10) in depression and stress, with lower scores in the physical than the executive function training group (Table 2). The Session effect suggested decreased depression and stress after training, *F*s ≥ 5.60, *p*s ≤ 0.023, η_p_^2^s ≥ 0.12. The interactions were not significant (*p*s ≥ 0.181).

The ANOVA on the IADL score did not reveal any significant effects (*p*s ≥ 0.423).

#### Summary

Taken together, results suggest that executive function training yielded positive cognitive near transfer effects to the WCST-64 relative to the physical training. Bayesian analysis also confirmed these transfer effects by showing a moderate to strong effect in three important variables of the WCST-64 (BF = 4.32–32.47). Additionally, depression and stress levels dropped following both training programs. Limited or no transfer effects were observed for any other cognitive or psychosocial outcome measures.

## Discussion

This study compared the cognitive and psychosocial benefits of executive function and low-intensity physical training programs in healthy older adults. Replicating previous findings (Wilkinson and Yang, 2012, 2016a; Yang et al., 2006), there was a significant practice effect on all executive function training tasks, validating the feasibility and efficacy of this program for eliciting practice effects. Compared to the low-intensity physical training program, executive function training also yielded positive, though limited, cognitive near transfer effects to the WCST-64, a measure of general executive control. No transfer effects were found to untrained cognitive abilities or daily living functions. Interestingly, depression and stress levels dropped following both training programs.

### Cognitive Transfer Effects

The current findings provide evidence that self-guided online executive function training can produce near transfer effects to a general executive control task (i.e., WCST-64) but little far transfer to untrained cognitive abilities. This is consistent with a recent meta-analysis showing that the effects of cognitive training tend to be specific and do not generalize to other real-world cognitive skills (Melby-Lervåg et al., 2016). In line with the literature (Karr et al., 2014; Sprague et al., 2019), the current study revealed cognitive near transfer effects in the executive function training group as compared to the physical training group. The benefits are unlikely accounted for by training time or overall engagement level. The analysis on the logged training time at each session (*n* = 13 in the executive function and *n* = 15 in the physical group without missing data) showed that the physical group spent more time in training than the executive function group, particularly in the first 5 weeks of training (*p*s ≤ 0.001). Furthermore, the self-reported weekly log of general cognitive and physical engagement outside of training, did not differ between the two groups or across weeks (*p*s ≥ 0.531). Thus, training time and general engagement likely did not contribute to the near transfer effect in the executive function group.

One recent meta-analysis found no transfer of CCT to executive functions (Lampit et al., 2014), whereas others showed selective transfer of CCT to shifting and inhibition but not updating abilities (Webb et al., 2018) in healthy older adults. Unlike these findings, the current study showed limited transfer to ability-specific pure executive function tasks (i.e., N-Back, Stroop, Navon, and Change-Detection). One possibility is that the ability-specific nature of CCT in previous work may only elicit ability-specific benefits. The online executive function training used in the current study differs from CCT used in previous work in its accessibility, adaptability, and complexity. Most selected training tasks (e.g., *Route to Sprout* or *Pinball Recall*) engaged multiple executive functions or working memory skills. These features of the executive function training program in the current study may explain its limited or lack of transfer to ability-specific executive function tasks. It should be noted that both training programs improved performance on those less-challenging conditions (the 1-back block in the *N-*back task and the 1-target condition of the Change Detection Task), which may simply reflect a retest practice effect. Further research with a waitlist no-training control group is needed to rule out the retest practice effect.

Additionally, the transfer effects to the WCST-64 somewhat support transfers based on training abilities rather than task structure. The WCST-64 requires inferring and updating the sorting criteria (i.e., color, shape, and number of symbols) based on response feedback. The “Brain Shift” and “Disillusion” training tasks also require monitoring and shifting between response rules, but the rules were pre-set and provided to participants. Thus, the demonstrated near transfer to the WCST-64 likely suggests a general near transfer effect to the trained ability beyond the specific task structure, extending the results of non-item-specific retest learning (Yang et al., 2009). Additionally, the WCST-64 requires participants to continuously plan, reason, and update task rules and to map the rules to four motor key-pressing response options. Thus, responses to the WCST-64 task may be more dependent on manual dexterity (i.e., coordinated fine motor skills), which is strongly related to executive function in older adults (Kobayashi-Cuya et al., 2018). This may also account for the near transfer to the WCST-64.

No cognitive far transfer was detected to speed (DSST) or memory (HVLT-R). This finding is consistent with literature showing minimal or no cognitive far transfer effects following CCT (Yang et al., 2006; Owen et al., 2010; Wilkinson and Yang, 2016a), presumably because the training involves repeatedly practicing the same set of cognitive skills without learning any other new skills. Consistent with this idea, it has been found that age-related decline in complex cognition, such as reasoning and episodic memory, cannot be explained by executive function differences (Verhaeghen, 2011). However, the lack of the cognitive far transfer effects is inconsistent with some other studies (Zelinski, 2009; Jaeggi et al., 2010; Hindin and Zelinski, 2012). Further research is thus needed to identify factors underlying the previously reported cognitive far transfer effects.

### Psychosocial Transfer Effects

Depression and stress levels dropped following both cognitive and physical training in the current study. These findings are consistent with earlier work showing reduced depression risk following cognitive training (Wolinsky et al., 2009) and alleviated depression following physical exercise (Bridle et al., 2012) and may suggest that exercise, whether cognitive or physical, has the potential to enhance certain psychosocial functions. We should note, however, that this benefit could be merely driven by a decrease in stress or excitement related to performing the training or transfer tasks over time. Again, given the exploratory nature of our study, further research with a no-training control group would help to rule out accounts related to reduced stress or excitement over time.

The lack of benefits on the self-reported IADL may be due to low variability and a ceiling effect, with an average score of 7.70–7.8 out of 8 across sessions and groups (Table 2). Nevertheless, the results are consistent with the ACTIVE study which found no immediate benefit on self-reported IADLs within the first 3 years of cognitive training, despite a long-term benefit after a 5–10-year delay (Rebok et al., 2014). However, we should note that far transfer to everyday functions (including a timed IADL task) has been previously found with community-dwelling older adults following practice of a useful field of view task in a meta-analysis (Edwards et al., 2018).

Inconsistent with the literature (Hillman et al., 2008; Smith et al., 2010) and our hypothesis, physical training in this study produced little benefit. The improvement was expressed only on stress, depression, and two easy cognitive task conditions (the 1-back block in the *N-*back task and the 1-target condition of the Change Detection Task). This might be due to the low-intensity nature of the training (HRR = 40.21%; Kramer et al., 2002), though training was well maintained across sessions. Given the self-guided and home-based nature of training, we prioritized participants’ safety and feasibility by choosing age-appropriate workouts (Nielsen et al., 2019), but they may not have been intensive enough to achieve the target HRR (e.g., 50–75%). Additionally, while executive function training was adapted to individual performance levels, the physical training group only had three options and thus it was not individually tailored. This may have restricted the potential benefits of the physical training program.

### Limitations and Outlook

A number of limitations should be noted. First, the small sample size might have limited our ability to detect subtle effects. Although the power analysis indicated that our sample size had 85% power to detect a moderate Group × Session interaction (*f* = 0.25), caution should be taken in interpreting the transfer results (specifically those non-significant effects) based on merely hypothesis testing results. Second, despite random assignment, the physical group showed an overall better cognitive/psychosocial profile than the executive function group at baseline (on DSST, depression, stress, WCST-64 total correct and conceptual level responses, *p*s ≤ 0.048). The smaller benefit of the physical group might be due to their higher baseline functioning, leaving little room for further improvement relative to the executive function group. However, it is important to note that the reported transfer effects (i.e., to the WCST-64) remained significant after the baseline group differences were controlled for in the analyses based on the proportional training gains and artificially baseline-matched samples. Furthermore, Bayesian analyses also confirmed the robustness of the transfer effects to the WCST-64. Third, the low-intensity nature of the physical exercise program might have restricted its potential beneficial effects, considering that some prior work has suggested that the cognitive benefits of physical exercise are intensity-dependent (Angevaren et al., 2007; Northey et al., 2018). Fourth, it is a challenge to make a fair comparison between the two programs as it is difficult to match the programs based on critical variables such as intensity level and quantitative outcome variables (e.g., behavioral index vs. heart rate change). To circumvent these challenges, we controlled for other important variables including the frequency and engagement time of the training sessions. As such, the results should be interpreted with caution in light of these challenges. Lastly, like some previous large-scale training studies (ACTIVE for example), the current study did not include an active or passive no-training control group to rule out retest practice effects. Nevertheless, the current study took an exploratory first step to evaluating and comparing the feasibility of using self-guided executive function and physical training programs with healthy older adults. Our results suggest a differential benefit of executive function training on a near transfer test of cognition, relative to the physical training group. The current study therefore adds value to the literature on behavioral interventions for improving older adults’ cognition by highlighting potential differences in the effects of cognitive and physical training.

## Conclusion

The results of the current study expand previous evidence for the efficacy of CCT in healthy older adults (Lampit et al., 2014). Specifically, the findings provide evidence, though limited, for the feasibility and efficacy of online executive function training for improving general executive function performance over and above a low-intensity physical exercise program in healthy older adults. These results add to the self-guided practice/training literature (Yang, 2011; Hindin and Zelinski, 2012) by validating that older adults can engage and adhere to a self-guided training program at home. Future research is required to identify the mechanisms underlying these transfer effects and to determine what factors may enhance the motivation and commitment of older adults to self-guided online cognitive training programs and thus maximize training benefits. For example, using multi-domain or combined training programs may be promising for far transfer effects to everyday functions.

## Disclosure Statement

We confirm that all the behavior measures, manipulation conditions, data inclusion and exclusion procedures, and sample size determination approach involved in this study were reported.

## Data Availability Statement

The datasets generated in this study can be found in online repositories. The names of the repository/repositories and accession number(s) can be found below: the data files and the related data file catalog file were deposited for open access to the Open Science Framework (https://osf.io/d7qrj/).

## Ethics Statement

The project received ethics approval from the Ryerson Ethics Board (REB2013-061). The participants provided their written informed consent to participate in this study.

## Author Contributions

LY directed the design and operation of the project, conducted data analyses, and led the manuscript preparation. SG and LW were involved in the actual operation of the project, including selecting and validating the physical training programs, selecting games for the executive function training program, training the research assistants, preparing testing packages, etc. BD was involved in programming and developing some cognitive outcome measures. All the authors contributed to the reviewing and editing of the earlier versions of the manuscript.

## Conflict of Interest

The authors declare that the research was conducted in the absence of any commercial or financial relationships that could be construed as a potential conflict of interest.

## Footnotes

1 http://www.lumosity.com

## References

[B1] Angevaren M. Vanhees L. Wendel-Vos W. Verhaar H. J. J. Aufdemkampe G. Aleman A. (2007). Intensity, but not duration, of physical activities is related to cognitive function. *Eur. J. Cardiovasc. Prev. Rehabil* 14 825–830. 10.1097/HJR.0b013e3282ef995b 18043306

[B2] Ansai J. H. Rebelatto J. R. (2015). Effect of two physical exercise protocols on cognition and depressive symptoms in oldest-old people: a randomized controlled trial. *Geriatr. Gerontol. Int*. 15 1127–1134. 10.1111/ggi.12411 25407380

[B3] Ball K. Berch D. B. Helmers K. F. Jobe J. B. Leveck M. D. Marsiske M. (2002). Effects of cognitive training interventions with older adults: a randomized controlled trial. *JAMA* 288 2271–2281. 10.1001/jama.288.18.2271 12425704 PMC2916176

[B4] Ballesteros S. Prieto A. Mayas J. Toril P. Pita C. Ponce de León L. (2015). Corrigendum: brain training with non-action video games enhances aspects of cognition in older adults: a randomized controlled trial. *Front. Aging Neurosci*. 7:82. 10.3389/fnagi.2015.00082 26042032 PMC4436590

[B5] Benedict H. B. R. Hopkins B. J. (2020). *Hopkins Verbal Learning Test–Revised.* Available online at: https://www.parinc.com/Products/Pkey/130 (accessed September 2, 2020).

[B6] Berryman N. Bherer L. Nadeau S. Lauzière S. Lehr L. Bobeuf F. (2014). Multiple roads lead to Rome: combined high-intensity aerobic and strength training vs. gross motor activities leads to equivalent improvement in executive functions in a cohort of healthy older adults. *AGE* 36:9710. 10.1007/s11357-014-9710-8 25194940 PMC4156938

[B7] Bridle C. Spanjers K. Patel S. Atherton N. M. Lamb S. E. (2012). Effect of exercise on depression severity in older people: systematic review and meta-analysis of randomised controlled trials. *Br. J. Psychiatry*. 201 180–185. 10.1192/bjp.bp.111.095174 22945926

[B8] CSTF (2020). *Fitness Canada. Canadian Standardized Test of Fitness (CSTF) : Operations Manual. Fitness and Amateur Sport; 1986*, 3 Edn Available online at: https://www.topendsports.com/testing/tests/step-canadian.htm (accessed September 2, 2020).

[B9] Edwards J. D. Fausto B. A. Tetlow A. M. Corona R. T. Valdés E. G. (2018). Systematic review and meta-analyses of useful field of view cognitive training. *Neurosci. Biobehav. Rev*. 84 72–91. 10.1016/j.neubiorev.2017.11.004 29175362

[B10] Faul F. Erdfelder E. Lang A.-G. Buchner A. (2007). G^∗^Power 3: a flexible statistical power analysis program for the social, behavioral, and biomedical sciences. *Behav. Res. Methods* 39 175–191. 10.3758/BF03193146 17695343

[B11] Folstein M. F. Folstein S. E. McHugh P. R. (1975). “Mini-mental state”. A practical method for grading the cognitive state of patients for the clinician. *J. Psychiatric Res*. 12 189–198. 10.1016/0022-3956(75)90026-6 1202204

[B12] Grady C. (2012). The cognitive neuroscience of ageing. *Nat. Rev. Neurosci*. 13 491–505. 10.1038/nrn3256 22714020 PMC3800175

[B13] Green C. S. Bavelier D. Kramer A. F. Vinogradov S. Ansorge U. Ball K. (2019). Improving methodological standards in behavioral interventions for cognitive enhancement. *J. Cogn. Enhanc*. 3 2–29. 10.1007/s41465-018-0115-y

[B14] Hardy J. L. Drescher D. Sarker K. Kellett G. Scanlon M. (2011). Enhancing visual attention and working memory with a Web-based cognitive training program. *Mensa Res. J*. 2011 709–744.

[B15] Hillman C. H. Erickson K. I. Kramer A. F. (2008). Be smart, exercise your heart: exercise effects on brain and cognition. *Nat. Rev. Neurosci*. 9 58–65. 10.1038/nrn2298 18094706

[B16] Hindin S. B. Zelinski E. M. (2012). Extended practice and aerobic exercise interventions benefit untrained cognitive outcomes in older adults: a meta-analysis. *J. Am. Geriatr. Soc*. 60 136–141. 10.1111/j.1532-5415.2011.03761.x 22150209 PMC4130646

[B17] Jaeggi S. M. Studer-Luethi B. Buschkuehl M. Su Y.-F. Jonides J. Perrig W. J. (2010). The relationship between n-back performance and matrix reasoning −– implications for training and transfer. *Intelligence* 38 625–635. 10.1016/j.intell.2010.09.001

[B18] Jost K. Bryck R. L. Vogel E. K. Mayr U. (2011). Are old adults just like low working memory young adults? Filtering efficiency and age differences in visual working memory. *Cereb Cortex*. 21 1147–1154. 10.1093/cercor/bhq185 20884722

[B19] Kable J. W. Caulfield M. K. Falcone M. McConnell M. Bernardo L. Parthasarathi T. (2017). No effect of commercial cognitive training on brain activity, choice behavior, or cognitive performance. *J. Neurosci*. 37 7390–7402. 10.1523/JNEUROSCI.2832-16.2017 28694338 PMC5546110

[B20] Karr J. E. Areshenkoff C. N. Rast P. Garcia-Barrera M. A. (2014). An empirical comparison of the therapeutic benefits of physical exercise and cognitive training on the executive functions of older adults: a meta-analysis of controlled trials. *Neuropsychology* 28 829–845. 10.1037/neu0000101 24933486

[B21] Kelly M. E. Loughrey D. Lawlor B. A. Robertson I. H. Walsh C. Brennan S. (2014). The impact of cognitive training and mental stimulation on cognitive and everyday functioning of healthy older adults: a systematic review and meta-analysis. *Ageing Res. Rev*. 15 28–43. 10.1016/j.arr.2014.02.004 24607830

[B22] Kobayashi-Cuya K. E. Sakurai R. Sakuma N. Sakuma N. Suzuki H. Yasunaga M. (2018). Hand dexterity, not handgrip strength, is associated with executive function in Japanese community-dwelling older adults: a cross-sectional study. *BMC Geriatr*. 18:192. 10.1186/s12877-018-0880-6 30143006 PMC6109297

[B23] Kongs K. S. Thompson L. L. Iverson G. L. Heaton R. K. (2000). *Wisconsin Card Sorting Test 64 Card Version (WCST-64). Psychological Assessment Research.* Available online at: https://www.parinc.com/Products/Pkey/479 (accessed September 2, 2020).

[B24] Kramer A. F. Colcombe S. Erickson K. Belopolsky A. McAuley E. Cohen N. J. (2002). Effects of aerobic fitness training on human cortical function. *J. Mol. Neurosci*. 19 227–231. 10.1007/s12031-002-0038-y 12212786

[B25] Kriska A. M. Pereira M. Fitzgerald S. Gregg S. (1997). Modifiable activity questionnaire in: a collection of physical activity questionnaires for health-related research. *Med. Sci. Sports Exerc.* 29(Suppl. 6) 73–78. 9243481

[B26] Krucoff C. (1990). *WHY JANE’S FONDA EXERCISE. Washington Post.* Available online at: https://www.washingtonpost.com/archive/lifestyle/wellness/1990/03/13/why-janes-fonda-exercise/25c54676-7574-4211-a950-89f22b522d1c/ (accessed September 2, 2020).

[B27] Lampit A. Hallock H. Valenzuela M. (2014). Computerized cognitive training in cognitively healthy older adults: a systematic review and meta-analysis of effect modifiers. *PLoS Med*. 11:1001756. 10.1371/journal.pmed.1001756 25405755 PMC4236015

[B28] Lawton M. P. Brody E. M. (1969). Assessment of older people: self-maintaining and instrumental activities of daily living. *Gerontologist* 9 179–186. 5349366

[B29] Lovibond P. F. Lovibond S. H. (1995). The structure of negative emotional states: comparison of the depression anxiety stress scales (DASS) with the beck depression and anxiety inventories. *Behav. Res. Therapy* 33 335–343. 10.1016/0005-7967(94)00075-U 7726811

[B30] Lustig C. Hasher L. Zacks R. T. (2007). “Inhibitory deficit theory: recent developments in a “new view”,” in *Inhibition in Cognition*, eds Gorfein D. S. MacLeod C. M. (Worcester, MA: American Psychological Association), 145–162. 10.1037/11587-008

[B31] Melby-Lervåg M. Redick T. S. Hulme C. (2016). Working memory training does not improve performance on measures of intelligence or other measures of “far transfer”: evidence from a meta-analytic review. *Perspect Psychol. Sci*. 11 512–534. 10.1177/1745691616635612 27474138 PMC4968033

[B32] Navon D. (1977). Forest before trees: the precedence of global features in visual perception. *Cogn. Psychol*. 9 353–383. 10.1016/0010-0285(77)90012-3

[B33] Nielsen T.-T. Møller T. K. Andersen L. L. Zebis M. K. Hansen P. R. Krustrup P. (2019). Feasibility and health effects of a 15-week combined exercise programme for sedentary elderly: a randomised controlled trial. *BioMed Res. Int.* 2019:3081029. 10.1155/2019/3081029 PMC6364114 30809536

[B34] Northey J. M. Cherbuin N. Pumpa K. L. Smee D. J. Rattray B. (2018). Exercise interventions for cognitive function in adults older than 50: a systematic review with meta-analysis. *Br. J. Sports Med*. 52 154–160. 10.1136/bjsports-2016-096587 28438770

[B35] Osman A. Wong J. L. Bagge C. L. Freedenthal S. Gutierrez P. M. Lozano G. (2012). The depression anxiety stress scales—21 (DASS-21): further examination of dimensions, scale reliability, and correlates. *J. Clin. Psychol*. 68 1322–1338. 10.1002/jclp.21908 22930477

[B36] Owen A. M. Hampshire A. Grahn J. A. Stenton R. Dajani S. Burns A. S. (2010). Putting brain training to the test. *Nature* 465 775–778. 10.1038/nature09042 20407435 PMC2884087

[B37] Park D. C. Lodi-Smith J. Drew L. Haber S. Hebrank A. Bischof G. N. (2014). The impact of sustained engagement on cognitive function in older adults: the synapse project. *Psychol. Sci*. 25 103–112. 10.1177/0956797613499592 24214244 PMC4154531

[B38] Rebok G. W. Ball K. Guey L. T. Jones R. N. Kim H.-Y. King J. W. (2014). Ten-year effects of the advanced cognitive training for independent and vital elderly cognitive training trial on cognition and everyday functioning in older adults. *J. Am. Geriatr. Soc*. 62 16–24. 10.1111/jgs.12607 24417410 PMC4055506

[B39] Simons D. J. Boot W. R. Charness N. Gathercole S. E. Chabris C. F. Hambrick D. Z. (2016). Do “Brain-Training” programs work? *Psychol. Sci. Public Interest* 17 103–186. 10.1177/1529100616661983 27697851

[B40] Smith P. J. Blumenthal J. A. Hoffman B. M. Cooper H. Strauman T. A. Welsh-Bohmer K. (2010). Aerobic exercise and neurocognitive performance: a meta-analytic review of randomized controlled trials. *Psychosom Med*. 72 239–252. 10.1097/PSY.0b013e3181d14633 20223924 PMC2897704

[B41] Sprague B. N. Freed S. A. Webb C. E. Phillips C. B. Hyun J. Ross L. A. (2019). The impact of behavioral interventions on cognitive function in healthy older adults: a systematic review. *Ageing Res. Rev*. 52 32–52. 10.1016/j.arr.2019.04.002 31002885 PMC8830244

[B42] Stine-Morrow E. A. L. Parisi J. M. Morrow D. G. Park D. C. (2008). The effects of an engaged lifestyle on cognitive vitality: a field experiment. *Psychol. Aging* 23 778–786. 10.1037/a0014341 19140649 PMC2774272

[B43] Stroop J. (1935). Studies of interference in serial verbal reactions. *J. Exp. Psychol*. 18 643–662.

[B44] Swain R. A. Berggren K. L. Kerr A. L. Patel A. Peplinski C. Sikorski A. M. (2012). On Aerobic exercise and behavioral and neural plasticity. *Brain Sci*. 2 709–744. 10.3390/brainsci2040709 24961267 PMC4061809

[B45] Tse A. C. Y. Wong T. W. L. Lee P. H. (2015). Effect of low-intensity exercise on physical and cognitive health in older adults: a systematic review. *Sports Med. Open* 1:34. 10.1186/s40798-015-0034-8 26512340 PMC4612316

[B46] Van Doorn J. van den Bergh D. Bohm U. Dablander F. Derks K. Draws T. (2019). The JASP guidelines for conducting and reporting a bayesian analysis. *PsyArXiv.* [Preprint]. 10.31234/osf.io/yqxfr PMC8219590 33037582

[B47] Verhaeghen P. (2011). Aging and executive control: reports of a demise greatly exaggerated. *Curr. Dir. Psychol. Sci*. 20 174–180. 10.1177/0963721411408772 25866452 PMC4389903

[B48] Warburton D. E. R. Bredin S. S. D. Jamnik V. K. Gledhill N. (2011). Validation of the PAR-Q+ and ePARmed-X+. *Health Fit. J. Can*. 4 38–46. 10.14288/hfjc.v4i2.151

[B49] Webb S. L. Loh V. Lampit A. Bateman J. E. Birney D. P. (2018). Meta-analysis of the effects of computerized cognitive training on executive functions: a cross-disciplinary taxonomy for classifying outcome cognitive factors. *Neuropsychol Rev*. 28 232–250. 10.1007/s11065-018-9374-8 29721646

[B50] Wechsler D. (1981). The psychometric tradition: developing the wechsler adult intelligence scale. *Contemp. Educ. Psychol.* 6, 82–85. 10.1016/0361-476X(81)90035-7

[B51] Wilkinson A. J. Yang L. (2012). Plasticity of inhibition in older adults: Retest practice and transfer effects. *Psychol. Aging* 27 606–615. 10.1037/a0025926 22182362

[B52] Wilkinson A. J. Yang L. (2015). *Cognitivie Plasticity. The Encyclopedia of Adulthood and Aging.* New York, NY: John Wiley & Sons, Inc, 1–5.

[B53] Wilkinson A. J. Yang L. (2016a). Inhibition plasticity in older adults: practice and transfer effects using a multiple task approach. *Neural Plasticity* 2016 1–12. 10.1155/2016/9696402 PMC4738960 26885407

[B54] Wilkinson A. J. Yang L. (2016b). Long-term maintenance of inhibition training effects in older adults: 1- and 3-year follow-up. *J. Gerontol Ser. B Psychol. Sci. Soc. Sci*. 71 622–629. 25573153 10.1093/geronb/gbu179

[B55] Wolinsky F. D. Mahncke H. W. Weg M. W. V. Martin R. Unverzagt F. W. Ball K. K. (2009). The ACTIVE cognitive training interventions and the onset of and recovery from suspected clinical depression. *J. Gerontol. B Psychol. Sci. Soc. Sci*. 64B 577–585. 10.1093/geronb/gbp061 19617456 PMC2728092

[B56] Yang L. (2011). Practice-oriented retest learning as the basic form of cognitive plasticity of the aging brain. *J. Aging Res.* 2001:407074. 10.4061/2011/407074 22132328 PMC3206383

[B57] Yang L. Krampe R. T. Baltes P. B. (2006). Basic forms of cognitive plasticity extended into the oldest-old: retest learning, age, and cognitive functioning. *Psychol. Aging* 21 372–378. 10.1037/0882-7974.21.2.372 16768581

[B58] Yang L. Reed M. Russo F. A. Wilkinson A. A. (2009). New look at retest learning in older adults: learning in the absence of item-specific effects. *J. Gerontol. Ser. B Psychol. Sci. Soc. Sci*. 64B 470–473. 10.1093/geronb/gbp040 19502572

[B59] Younan B. (2018). Cognitive functioning differences between physically active and sedentary older adults. *J. Alzheimers Dis. Rep*. 2 93–101. 10.3233/ADR-180053 30480252 PMC6159626

[B60] Zelinski E. M. (2009). Far transfer in cognitive training of older adults. *Restorat. Neurol. Neurosci*. 27 455–471. 10.3233/RNN-2009-0495 19847070 PMC4169295

