# Supplementary material for: Cognitive and Psychosocial Outcomes of Self-Guided Executive Function Training and Low-Intensity Aerobic Exercise in Healthy Older Adults
Source: Front Aging Neurosci. 2020 Nov 19;12:576744. doi: 10.3389/fnagi.2020.576744 (PMC7710606; doi:10.3389/fnagi.2020.576744)
Supplement: Supplementary file 1 [file Table_1.pdf]

Appendix A. Executive Function Training Tasks (i.e., Lumosity games)

Game (Session#)	Ability Domain	Task Description
Brain Shift (1-40)	Task Switching	Participants need to switch between two task rules depending on the location (top or bottom) of the letter-number pair, unpredictably, and determine (1) whether the number on the top card is even (e.g. 'M6', correct response: 'YES'), or (2) whether the letter on the bottom card is a vowel (e.g., 'K7', correct response: 'NO'). The task is adaptive, with rules present initially but absent later.
Colour Match (1-40)	Response Inhibition	Participants view two color words side by side and determine whether the *meaning* of the word on the left matches the *ink colour* of the word on the right (e.g., for the word "RED" on the left and the word "BLUE" printed in red ink on the right, the correct response would be "match")
Face Memory Workout (1-40)	Working Memory	A modified *n*-back task in which participants view sequentially presented male and female faces and determine whether the current face matches the one presented at the 1-back, 2-back, or 3-back position. The task is adaptive by progressively eliminating the previous faces from the viewing window and/or increasing the memory span (1-back gradually to 3-back).
Lost in Migration (1-40)	Selective Attention	Participants view a flock of birds appearing on the screen, with the central bird facing in the same or opposite direction from the surrounding birds. They need to determine the direction of the central bird regardless of the direction of the surrounding birds.
Memory Matrix (1-40)	Working Memory	Participants view a square grid, starting with 9 squares and 3 of which briefly turn dark maroon all at once, and need to click the squares to recreate the pattern. The task is adaptive by gradually increasing the size of the grid and/or the number of dark maroon tiles to be monitored.
Disillusion (10-40)	Task Switching	Participants quickly match a puzzle piece to a pattern with a missing piece by unpredictably switching between two rules, 1) matching a vertical piece to the pattern based on COLOUR (e.g., *red* circle to *red* square); or 2) matching a horizontal piece to the pattern based on SHAPE (e.g., blue *triangle* to green *triangle*).
Follow the Frog (15-40)	Working Memory	Participants follow and recreate the path of an orange frog as it jumps from one to another lily pad by clicking on the lily pads in the same order, as the frog continuously jumps.
Route to Sprout (20-40)	Planning, Problem Solving	Participants need to find and click the optimal route (with the least number of moves) to guide a seed to its planting hole, while moving aside the blocking obstacles (i.e., ladybugs). A timer starts after the first move.
Observation Tower (25-40)	Attention	Participants view digits briefly appearing in circles on the screen, remember and reproduce the locations of the digits, from smallest to largest, to construct the tallest tower.
Pinball Recall (30-40)	Working Memory	Participants need to memorize the locations of several bumpers before they disappear, then visualize how the pinball will bounce off them, and determine where it will go.
